# Supplementary figures and images for: Effects of probiotic supplements on growth performance and intestinal microbiota of partridge shank broiler chicks
Source: PeerJ. 2021 Dec 1;9:e12538. doi: 10.7717/peerj.12538 (PMC8643103; doi:10.7717/peerj.12538)

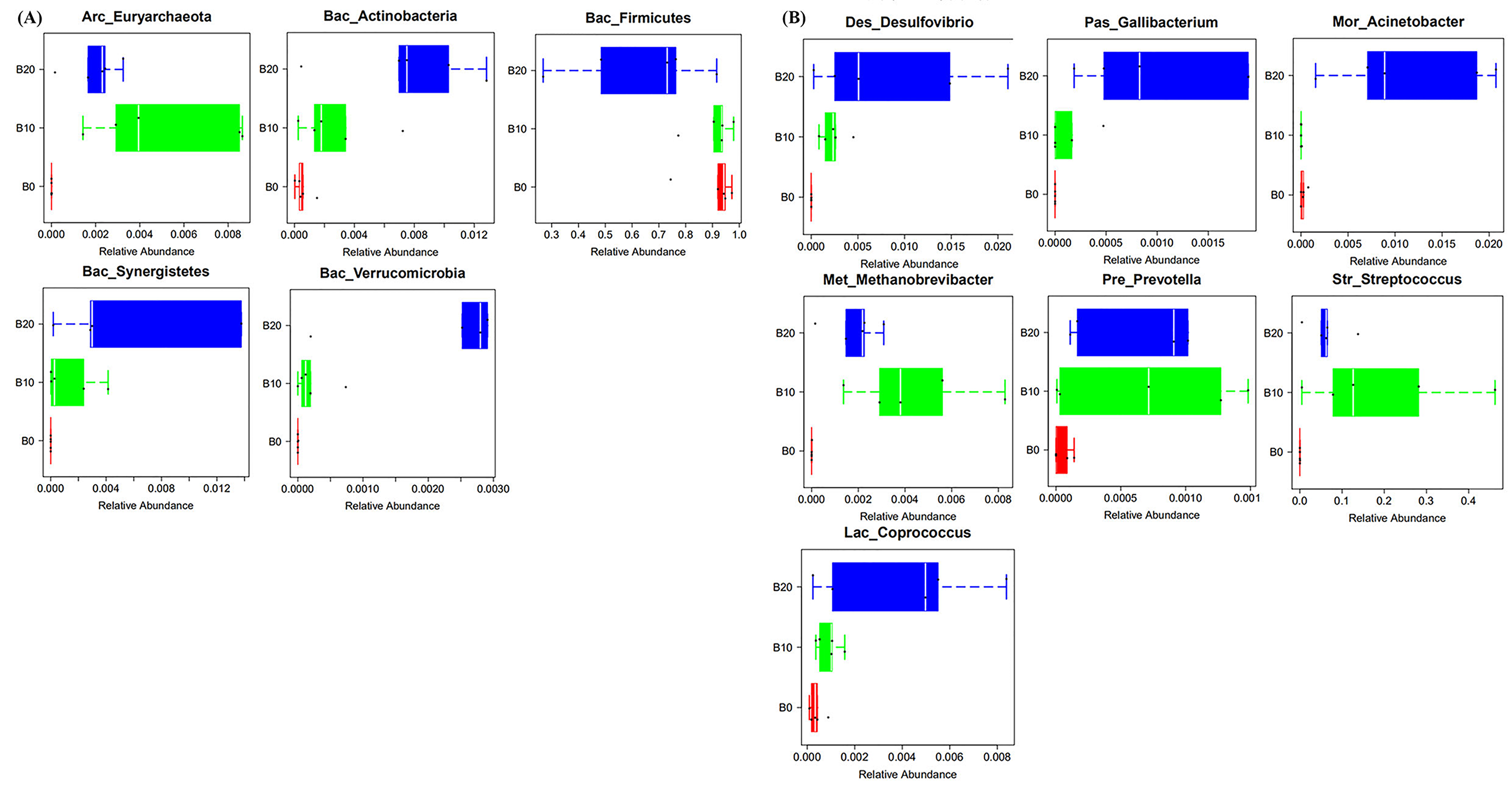

Supplement: Supplemental Information 1 [file peerj-09-12538-s001.png]

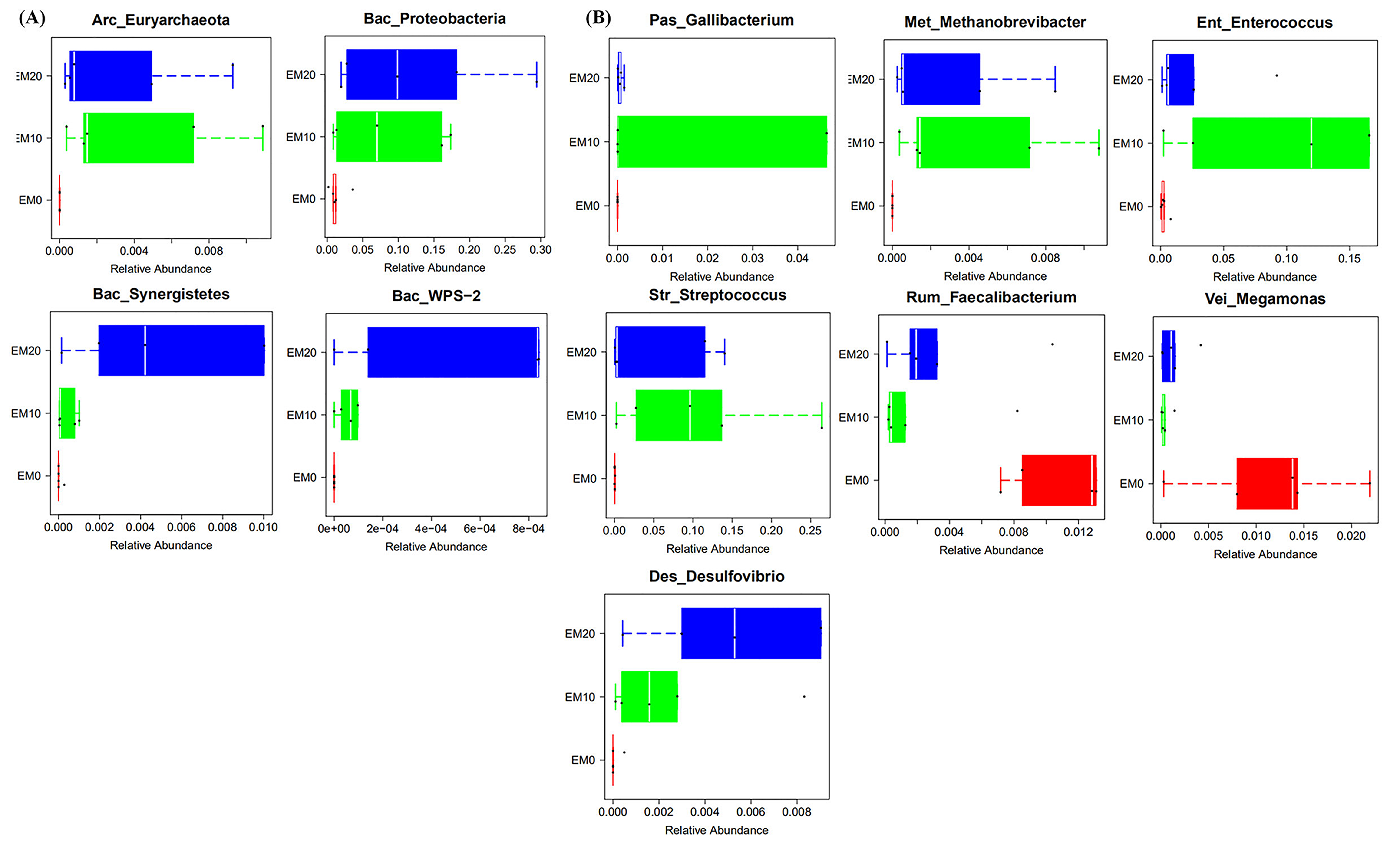

Supplement: Supplemental Information 2 [file peerj-09-12538-s002.png]

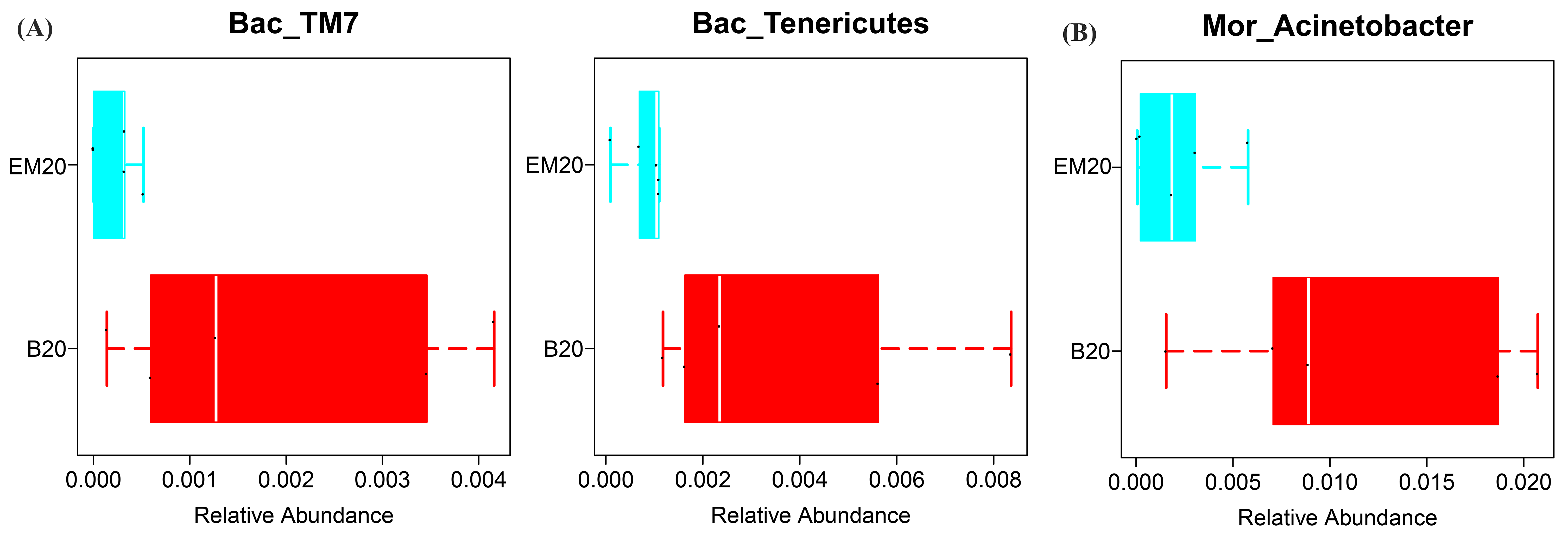

Supplement: Supplemental Information 3 [file peerj-09-12538-s003.png]

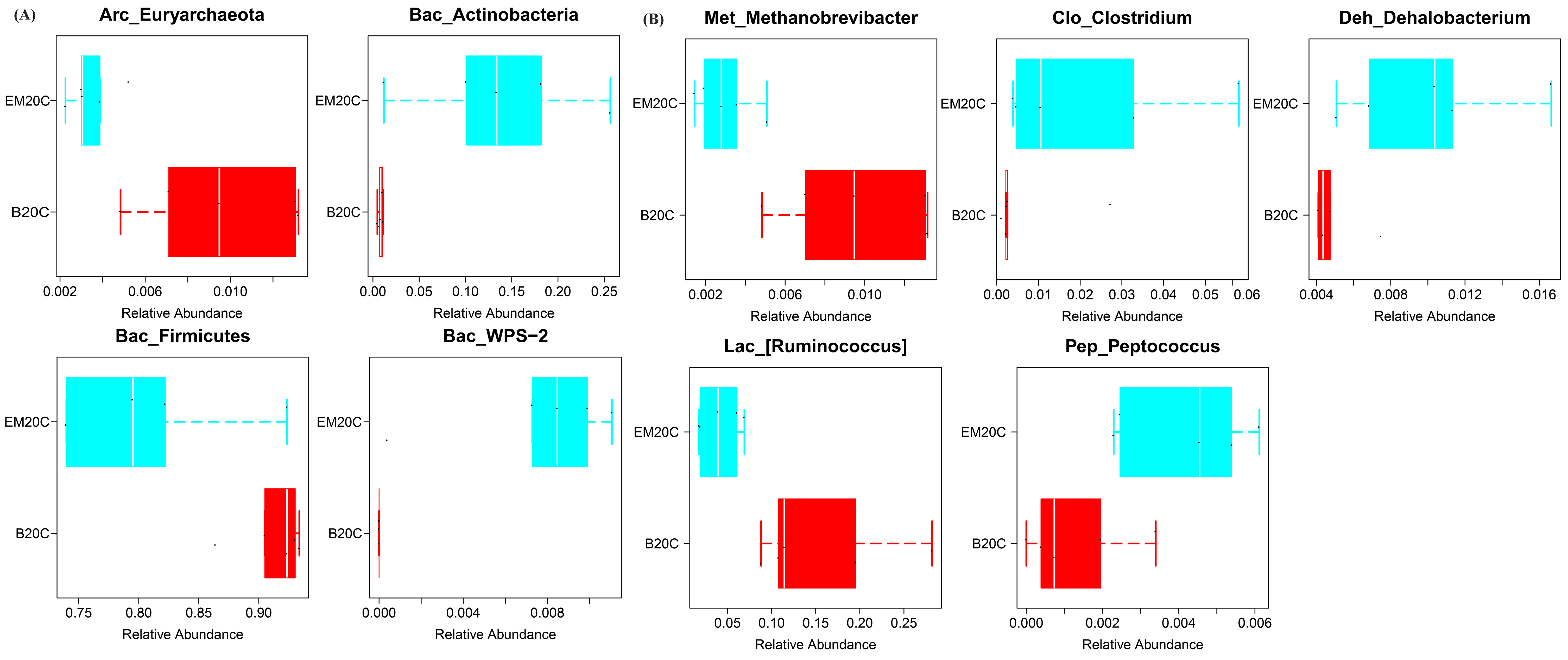

Supplement: Supplemental Information 4 [file peerj-09-12538-s004.png]
