# Supplementary material for: Effects of probiotic supplements on growth performance and intestinal microbiota of partridge shank broiler chicks
Source: PeerJ. 2021 Dec 1;9:e12538. doi: 10.7717/peerj.12538 (PMC8643103; doi:10.7717/peerj.12538)
Supplement: Supplemental Information 5 [file peerj-09-12538-s005.docx]

Table S1 Construction of EM used in this study sequenced by 16S rRNA

V3-V4 hypervariable regions on MiSeq Platform

| Phylum | Genus | Abundance, % | |
| --- | --- | --- | --- |
|  |  | Average | SD |
| *Firmicutes* | *Lactobacillus* | 84.021 | 12.309 |
|  | *Anoxybacillus* | 0.367 | 0.549 |
|  | *Lysinibacillus* | 0.207 | 0.314 |
|  | *Faecalibacterium* | 0.190 | 0.214 |
|  | *Roseburia* | 0.141 | 0.195 |
|  | *Phascolarctobacterium* | 0.115 | 0.184 |
|  | *Staphylococcus* | 0.078 | 0.127 |
|  | *Bacillus* | 0.092 | 0.115 |
|  | *Lachnospira* | 0.109 | 0.103 |
|  | *Brevibacillus* | 0.056 | 0.092 |
|  | *Streptococcus* | 0.057 | 0.080 |
|  | *Megamonas* | 0.059 | 0.056 |
| *Bacteroidetes* | *Bacteroides* | 1.374 | 1.887 |
|  | *Prevotella* | 0.130 | 0.218 |
|  | *Parabacteroides* | 0.131 | 0.200 |
| *Proteobacteria* | *Halomonas* | 0.680 | 1.108 |
|  | *Citrobacter* | 0.231 | 0.400 |
|  | *Ochrobactrum* | 0.180 | 0.296 |
|  | *Pseudomonas* | 0.139 | 0.239 |
|  | *Ralstonia* | 0.116 | 0.171 |
|  | *Sphingomonas* | 0.056 | 0.095 |
|  | *Agrobacterium* | 0.046 | 0.073 |
|  | *Acinetobacter* | 0.039 | 0.064 |
|  | *Acetobacter* | 5.297 | 9.169 |
| *[Thermi]* | *Meiothermus* | 0.853 | 1.444 |
| *Actinobacteria* | *Gordonia* | 0.193 | 0.307 |
|  | *Propionibacterium* | 0.109 | 0.169 |
|  | *Renibacterium* | 0.066 | 0.099 |
| Others/unassigned |  | 4.868 | 5.341 |
