# Supplementary material for: Effects of probiotic supplements on growth performance and intestinal microbiota of partridge shank broiler chicks
Source: PeerJ. 2021 Dec 1;9:e12538. doi: 10.7717/peerj.12538 (PMC8643103; doi:10.7717/peerj.12538)
Supplement: Supplemental Information 6 [file peerj-09-12538-s006.docx]

Table S2 Summary of sequencing data obtained in this study

| Group | sample | tags | singleton | singleton% | clean_tags | OTU |
| --- | --- | --- | --- | --- | --- | --- |
| B0 | B0_1 | 100360 | 91732 | 91.403 | 44071 | 401 |
|  | B0_2 | 81727 | 78618 | 96.196 | 44543 | 291 |
|  | B0_3 | 105166 | 63852 | 60.715 | 85953 | 489 |
|  | B0_4 | 69341 | 65790 | 94.879 | 42289 | 241 |
|  | B0_5 | 139994 | 9019 | 6.442 | 138263 | 297 |
| EM0 | EM0_1 | 136917 | 46872 | 34.234 | 130221 | 564 |
|  | EM0_2 | 139717 | 16498 | 11.808 | 137207 | 452 |
|  | EM0_3 | 139208 | 9247 | 6.643 | 137120 | 462 |
|  | EM0_4 | 84043 | 81957 | 97.518 | 55612 | 166 |
|  | EM0_5 | 74242 | 70941 | 95.554 | 61224 | 258 |
| B10 | B10_1 | 139764 | 10141 | 7.256 | 136512 | 610 |
|  | B10_2 | 66920 | 64378 | 96.201 | 55052 | 184 |
|  | B10_3 | 101894 | 28011 | 27.490 | 99531 | 421 |
|  | B10_4 | 109655 | 62738 | 57.214 | 103294 | 587 |
|  | B10_5 | 109700 | 8621 | 7.859 | 107729 | 622 |
| EM10 | EM10_1 | 139958 | 8506 | 6.078 | 138234 | 300 |
|  | EM10_2 | 104935 | 43717 | 41.661 | 102588 | 286 |
|  | EM10_3 | 99123 | 18542 | 18.706 | 96880 | 532 |
|  | EM10_4 | 136909 | 12189 | 8.903 | 133946 | 740 |
|  | EM10_5 | 125645 | 10381 | 8.262 | 123237 | 659 |
| B20 | B20_1 | 110752 | 15108 | 13.641 | 107474 | 884 |
|  | B20_2 | 139947 | 9020 | 6.445 | 137202 | 427 |
|  | B20_3 | 101940 | 17291 | 16.962 | 99019 | 780 |
|  | B20_4 | 137308 | 16847 | 12.269 | 134155 | 971 |
|  | B20_5 | 115341 | 11041 | 9.572 | 111032 | 816 |
| EM20 | EM20_1 | 139493 | 13599 | 9.749 | 134969 | 731 |
|  | EM20_2 | 139335 | 15245 | 10.941 | 132005 | 880 |
|  | EM20_3 | 138897 | 11765 | 8.470 | 136284 | 647 |
|  | EM20_4 | 139906 | 8437 | 6.030 | 136849 | 387 |
|  | EM20_5 | 136893 | 16109 | 11.768 | 132510 | 1035 |
| B20C | B20_1C | 137551 | 19528 | 14.197 | 133131 | 1020 |
|  | B20_2C | 137009 | 19042 | 13.898 | 132785 | 965 |
|  | B20_3C | 88260 | 86118 | 97.573 | 65583 | 362 |
|  | B20_4C | 72248 | 69685 | 96.452 | 54607 | 439 |
|  | B20_5C | 89363 | 87075 | 97.440 | 67914 | 347 |
| EM20C | EM20_1C | 104834 | 48988 | 46.729 | 96468 | 714 |
|  | EM20_2C | 126874 | 16084 | 12.677 | 122126 | 876 |
|  | EM20_3C | 138218 | 18179 | 13.152 | 132797 | 1027 |
|  | EM20_4C | 128792 | 16717 | 12.980 | 125195 | 983 |
|  | EM20_5C | 125306 | 18467 | 14.738 | 118712 | 1077 |
