# Supplementary material for: Effects of probiotic supplements on growth performance and intestinal microbiota of partridge shank broiler chicks
Source: PeerJ. 2021 Dec 1;9:e12538. doi: 10.7717/peerj.12538 (PMC8643103; doi:10.7717/peerj.12538)
Supplement: Supplemental Information 10 [file peerj-09-12538-s010.docx]

**Raw data: Table 1 Body weight of broiler in EM-treated and control groups**

| EM-treated group, g | | | | | Control group, g | | | | |
| --- | --- | --- | --- | --- | --- | --- | --- | --- | --- |
| No. | Repetition | 0d | 10d | 20d | No. | Repetition | 0d | 10d | 20d |
| 1 | Repetition 1 | 159.9 | 289.0 | 581.0 | 51 | Repetition 6 | 134.0 | 251.0 | 519.8 |
| 2 |  | 136.8 | 258.6 | 505.6 | 52 |  | 137.4 | 252.3 | 529.8 |
| 3 |  | 139.2 | 251.9 | 511.6 | 53 |  | 129.5 | 244.8 | 511.8 |
| 4 |  | 147.4 | 269.9 | 573.1 | 54 |  | 158.8 | 278.2 | 558.2 |
| 5 |  | 145.9 | 279.2 | 561.9 | 55 |  | 143.8 | 239.5 | 518.1 |
| 6 |  | 146.6 | 273.1 | 581.1 | 56 |  | 144.9 | 248.0 | 530.0 |
| 7 |  | 145.2 | 277.5 | 561.0 | 57 |  | 147.5 | 251.2 | 541.0 |
| 8 |  | 150.9 | 291.6 | 591.0 | 58 |  | 148.3 | 245.0 | 533.0 |
| 9 |  | 145.9 | 270.0 | 525.0 | 59 |  | 143.2 | 239.9 | 521.0 |
| 10 |  | 145.9 | 273.1 | 568.2 | 60 |  | 144.5 | 240.0 | 533.0 |
| 11 | Repetition 2 | 145.6 | 278.5 | 573.1 | 61 | Repetition 7 | 143.8 | 241.3 | 525.0 |
| 12 |  | 139.7 | 278.5 | 595.0 | 62 |  | 147.0 | 275.0 | 555.0 |
| 13 |  | 149.7 | 281.0 | 588.2 | 63 |  | 148.8 | 255.3 | 548.3 |
| 14 |  | 144.0 | 267.7 | 571.2 | 64 |  | 151.2 | 269.0 | 549.8 |
| 15 |  | 144.8 | 270.1 | 577.2 | 65 |  | 147.6 | 255.8 | 539.0 |
| 16 |  | 143.4 | 261.5 | 511.9 | 66 |  | 146.7 | 255.0 | 536.0 |
| 17 |  | 146.8 | 271.5 | 556.8 | 67 |  | 147.5 | 256.9 | 551.0 |
| 18 |  | 147.4 | 271.0 | 571.9 | 68 |  | 146.5 | 259.1 | 540.0 |
| 19 |  | 139.8 | 271.0 | 571.2 | 69 |  | 145.3 | 248.2 | 518.0 |
| 20 |  | 144.2 | 270.2 | 571.1 | 70 |  | 145.5 | 248.0 | 522.0 |
| 21 | Repetition 3 | 143.5 | 270.3 | 581.2 | 71 | Repetition 8 | 147.5 | 241.6 | 509.2 |
| 22 |  | 143.0 | 264.8 | 528.9 | 72 |  | 148.0 | 248.2 | 529.0 |
| 23 |  | 144.6 | 269.7 | 559.0 | 73 |  | 133.0 | 239.0 | 508.2 |
| 24 |  | 131.7 | 261.2 | 548.9 | 74 |  | 148.0 | 261.3 | 536.0 |
| 25 |  | 129.9 | 255.8 | 506.3 | 75 |  | 142.0 | 244.0 | 513.0 |
| 26 |  | 144.0 | 263.9 | 521.0 | 76 |  | 145.3 | 258.2 | 541.0 |
| 27 |  | 155.1 | 281.5 | 579.2 | 77 |  | 143.9 | 256.3 | 521.0 |
| 28 |  | 148.6 | 271.0 | 577.0 | 78 |  | 152.0 | 269.1 | 551.2 |
| 29 |  | 158.9 | 294.0 | 581.2 | 79 |  | 155.8 | 270.1 | 562.3 |
| 30 |  | 143.8 | 280.1 | 571.3 | 80 |  | 142.5 | 238.1 | 501.2 |
| 31 | Repetition 4 | 143.5 | 269.4 | 521.2 | 81 | Repetition 9 | 148.3 | 256.8 | 526.0 |
| 32 |  | 142.7 | 265.0 | 520.6 | 82 |  | 143.3 | 236.0 | 499.5 |
| 33 |  | 143.9 | 264.9 | 502.3 | 83 |  | 152.0 | 268.2 | 550.8 |
| 34 |  | 137.9 | 261.3 | 521.0 | 84 |  | 147.1 | 241.2 | 513.2 |
| 35 |  | 147.1 | 279.5 | 581.6 | 85 |  | 144.6 | 255.3 | 522.0 |
| 36 |  | 147.4 | 277.8 | 581.8 | 86 |  | 158.1 | 281.3 | 576.9 |
| 37 |  | 133.8 | 255.9 | 512.3 | 87 |  | 146.9 | 253.9 | 526.0 |
| 38 |  | 155.7 | 291.5 | 601.1 | 88 |  | 142.0 | 241.8 | 512.5 |
| 39 |  | 150.4 | 281.0 | 602.5 | 89 |  | 147.7 | 271.0 | 561.2 |
| 40 |  | 148.3 | 292.1 | 606.2 | 90 |  | 146.6 | 261.3 | 551.2 |
| 41 | Repetition 5 | 144.8 | 285.6 | 603.5 | 91 | Repetition 10 | 145.0 | 239.2 | 509.2 |
| 42 |  | 143.8 | 275.3 | 568.2 | 92 |  | 148.9 | 271.9 | 539.2 |
| 43 |  | 159.1 | 295.1 | 614.1 | 93 |  | 152.0 | 271.8 | 539.5 |
| 44 |  | 146.0 | 277.1 | 577.5 | 94 |  | 146.0 | 251.2 | 528.2 |
| 45 |  | 155.8 | 288.5 | 609.1 | 95 |  | 146.2 | 255.6 | 533.2 |
| 46 |  | 146.8 | 281.5 | 563.2 | 96 |  | 146.9 | 254.0 | 533.2 |
| 47 |  | 160.2 | 290.1 | 616.2 | 97 |  | 141.9 | 234.5 | 501.2 |
| 48 |  | 133.8 | 257.9 | 512.8 | 98 |  | 149.8 | 280.0 | 590.8 |
| 49 |  | 144.8 | 278.5 | 576.3 | 99 |  | 146.7 | 261.3 | 539.5 |
| 50 |  | 146.0 | 279.8 | 577.0 | 100 |  | 146.4 | 264.2 | 532.9 |

**Raw data: Table 2 Average daily intake of broiler in each repetition**

| Experiment day | Repetition | | | | | | | | | |
| --- | --- | --- | --- | --- | --- | --- | --- | --- | --- | --- |
|  | EM-treated goup, g | | | | | Control group, g | | | | |
|  | 1 | 2 | 3 | 4 | 5 | 6 | 7 | 8 | 9 | 10 |
| day1 | 285 | 295 | 287 | 277 | 278 | 299 | 301 | 281 | 297 | 290 |
| day2 | 271 | 279 | 281 | 291 | 280 | 280 | 289 | 271 | 287 | 283 |
| day3 | 289 | 281 | 271 | 288 | 281 | 299 | 295 | 281 | 288 | 289 |
| day4 | 279 | 277 | 282 | 301 | 283 | 285 | 288 | 269 | 284 | 288 |
| day5 | 301 | 295 | 301 | 318 | 315 | 349 | 351 | 329 | 331 | 346 |
| day6 | 371 | 363 | 385 | 393 | 390 | 371 | 373 | 362 | 351 | 361 |
| day7 | 416 | 427 | 461 | 451 | 457 | 412 | 431 | 399 | 383 | 401 |
| day8 | 431 | 441 | 479 | 488 | 479 | 423 | 437 | 419 | 419 | 412 |
| day9 | 428 | 437 | 489 | 487 | 485 | 420 | 446 | 420 | 416 | 412 |
| day10 | 419 | 427 | 481 | 487 | 490 | 451 | 471 | 433 | 412 | 449 |
| day11 | 441 | 451 | 505 | 507 | 506 | 467 | 481 | 439 | 451 | 464 |
| day12 | 456 | 468 | 509 | 510 | 517 | 471 | 487 | 439 | 451 | 456 |
| day13 | 472 | 481 | 515 | 526 | 542 | 526 | 531 | 519 | 521 | 511 |
| day14 | 511 | 528 | 544 | 559 | 568 | 489 | 492 | 481 | 478 | 472 |
| day15 | 500 | 502 | 528 | 547 | 531 | 481 | 499 | 467 | 481 | 486 |
| day16 | 523 | 537 | 571 | 581 | 596 | 512 | 526 | 510 | 511 | 519 |
| day17 | 522 | 529 | 568 | 590 | 605 | 521 | 539 | 509 | 517 | 530 |
| day18 | 621 | 644 | 667 | 688 | 696 | 648 | 667 | 632 | 623 | 648 |
| day19 | 600 | 622 | 645 | 666 | 669 | 619 | 633 | 612 | 621 | 621 |
| day20 | 655 | 668 | 712 | 723 | 746 | 618 | 646 | 609 | 626 | 636 |

**Raw data: Table 3 The villus height and crypt depth of jejunum in chickens between EM-treated and control groups**

| broiler | group | repeat 1 | repeat 2 | repeat 3 | repeat 4 |
| --- | --- | --- | --- | --- | --- |
| villus height, µm | | | | | |
| 2 | EM-treated | 603.87 | 656.39 | 588.39 | 631.06 |
| 13 |  | 621.46 | 599.52 | 522.64 | 433.64 |
| 22 |  | 583.24 | 582.58 | 609.88 | 642.33 |
| 31 |  | 588.75 | 599.35 | 609.99 | 560.86 |
| 48 |  | 515.25 | 466.36 | 494.07 | 597.39 |
| 56 | Control | 34.45 | 35.65 | 28.38 | 39.66 |
| 62 |  | 44.88 | 36.75 | 27.95 | 37.71 |
| 77 |  | 58.02 | 45.43 | 49.02 | 52.03 |
| 83 |  | 35.64 | 64.11 | 26.72 | 28.25 |
| 94 |  | 39.55 | 36.47 | 43.99 | 26.25 |
|  | | | | | |
| crypt depth, µm | | | | | |
| 2 | EM-treated | 408.23 | 430.38 | 496.88 | 333.21 |
| 13 |  | 406.01 | 420.95 | 490.29 | 482.24 |
| 22 |  | 457.37 | 456.50 | 437.57 | 301.77 |
| 31 |  | 348.09 | 393.79 | 454.45 | 469.79 |
| 48 |  | 464.51 | 469.14 | 400.60 | 423.78 |
| 56 | Control | 40.90 | 38.82 | 38.21 | 50.78 |
| 62 |  | 54.12 | 74.88 | 55.89 | 77.23 |
| 77 |  | 52.89 | 40.13 | 42.00 | 53.94 |
| 83 |  | 42.19 | 42.03 | 64.02 | 63.95 |
| 94 |  | 58.09 | 46.57 | 65.64 | 46.06 |

**Raw data: Table 4 The villus height and crypt depth of ileum in chickens between EM-treated and control groups**

| Broiler | group | repeat 1 | repeat 2 | repeat 3 | repeat 4 |
| --- | --- | --- | --- | --- | --- |
| villus height, µm | | | | | |
| 2 | EM-treated | 582.92 | 586.52 | 492.07 | 511.87 |
| 13 |  | 523.33 | 476.01 | 483.84 | 561.81 |
| 22 |  | 450.95 | 463.85 | 466.23 | 491.62 |
| 31 |  | 499.42 | 505.38 | 513.09 | 578.98 |
| 48 |  | 546.73 | 552.91 | 543.46 | 571.59 |
| 56 | Control | 316.67 | 376.90 | 370.98 | 344.87 |
| 62 |  | 352.88 | 341.65 | 366.22 | 332.49 |
| 77 |  | 362.69 | 346.19 | 366.20 | 346.89 |
| 83 |  | 323.63 | 294.70 | 338.03 | 372.03 |
| 94 |  | 314.99 | 325.58 | 318.57 | 343.60 |
|  | | | | | |
| crypt depth, µm | | | | | |
| 2 | EM-treated | 36.90 | 37.68 | 40.57 | 42.20 |
| 13 |  | 43.87 | 24.83 | 20.92 | 42.11 |
| 22 |  | 27.81 | 24.06 | 33.54 | 32.16 |
| 31 |  | 36.33 | 28.00 | 34.90 | 16.33 |
| 48 |  | 26.72 | 47.09 | 55.04 | 50.22 |
| 56 | Control | 45.19 | 47.04 | 49.71 | 43.01 |
| 62 |  | 38.49 | 45.93 | 29.92 | 33.32 |
| 77 |  | 40.08 | 42.69 | 32.74 | 23.95 |
| 83 |  | 35.22 | 55.92 | 40.63 | 38.99 |
| 94 |  | 30.89 | 55.11 | 64.90 | 46.92 |

**Raw data: Table 3 The villus height and crypt depth of cecum in chickens between EM-treated and control groups**

| Broiler | group | repeat 1 | repeat 2 | repeat 3 | repeat 4 |
| --- | --- | --- | --- | --- | --- |
| villus height, µm | | | | | |
| 2 | EM-treated | 92.50 | 67.60 | 69.30 | 81.54 |
| 13 |  | 89.48 | 50.17 | 68.88 | 90.17 |
| 22 |  | 69.44 | 95.30 | 89.78 | 58.21 |
| 31 |  | 92.10 | 82.98 | 116.32 | 98.07 |
| 48 |  | 62.06 | 88.45 | 113.39 | 80.86 |
| 56 | Control | 76.98 | 40.53 | 54.03 | 61.20 |
| 62 |  | 54.42 | 50.69 | 50.95 | 43.33 |
| 77 |  | 59.38 | 47.39 | 44.79 | 41.51 |
| 83 |  | 41.80 | 58.46 | 42.00 | 50.65 |
| 94 |  | 56.88 | 46.01 | 50.04 | 37.71 |
|  | | | | | |
| crypt depth, µm | | | | | |
| 2 | EM-treated | 14.52 | 20.92 | 15.27 | 18.23 |
| 13 |  | 17.12 | 21.34 | 21.52 | 19.70 |
| 22 |  | 26.41 | 17.87 | 19.38 | 14.96 |
| 31 |  | 23.32 | 15.87 | 20.28 | 19.48 |
| 48 |  | 19.17 | 19.38 | 17.52 | 17.96 |
| 56 | Control | 19.36 | 29.93 | 29.48 | 17.95 |
| 62 |  | 23.02 | 16.28 | 26.26 | 19.60 |
| 77 |  | 24.74 | 20.66 | 14.57 | 22.71 |
| 83 |  | 22.62 | 21.24 | 24.20 | 12.70 |
| 94 |  | 21.07 | 29.06 | 28.00 | 24.91 |
